# Development of a Distance Education Program by a Land-Grant University Augments the 2-Year to 4-Year STEM Pipeline and Increases Diversity in STEM

**DOI:** 10.1371/journal.pone.0119548

**Published:** 2015-04-15

**Authors:** Jennifer C. Drew, Monika W. Oli, Kelly C. Rice, Alexandria N. Ardissone, Sebastian Galindo-Gonzalez, Pablo R. Sacasa, Heather J. Belmont, Allen F. Wysocki, Mark Rieger, Eric W. Triplett

**Affiliations:** 1 Microbiology and Cell Science Department, Institute of Food and Agricultural Sciences, University of Florida, Gainesville, Florida, United States of America; 2 Agricultural Education and Communications Department, Institute of Food and Agricultural Sciences, University of Florida, Gainesville, Florida, United States of America; 3 Miami Dade College, North Campus, Miami, Florida, United States of America; 4 Miami Dade College, School of Science, Miami, Florida, United States of America; 5 College of Agricultural and Life Sciences, Institute of Food and Agricultural Sciences, University of Florida, Gainesville, Florida, United States of America

## Abstract

Although initial interest in science, technology, engineering and mathematics (STEM) is high, recruitment and retention remains a challenge, and some populations are disproportionately underrepresented in STEM fields. To address these challenges, the Microbiology and Cell Science Department in the College of Agricultural and Life Sciences at the University of Florida has developed an innovative 2+2 degree program. Typical 2+2 programs begin with a student earning an associate’s degree at a local community college and then transferring to a 4-year institution to complete a bachelor’s degree. However, many universities in the United States, particularly land-grant universities, are located in rural regions that are distantly located from their respective states’ highly populated urban centers. This geographical and cultural distance could be an impediment to recruiting otherwise highly qualified and diverse students. Here, a new model of a 2+2 program is described that uses distance education as the vehicle to bring a research-intensive university’s life sciences curriculum to students rather than the oft-tried model of a university attempting to recruit underrepresented minority students to its location. In this paradigm, community college graduates transfer into the Microbiology and Cell Science program as distance education students to complete their Bachelor of Science degree. The distance education students’ experiences are similar to the on-campus students’ experiences in that both groups of students take the same department courses taught by the same instructors, take required laboratory courses in a face-to-face format, take only proctored exams, and have the same availability to instructors. Data suggests that a hybrid online transfer program may be a viable approach to increasing STEM participation (as defined by enrollment) and diversity. This approach is particularly compelling as the distance education cohort has comparable grade point averages and retention rates compared to the corresponding on-campus transfer cohort.

## Introduction

In 2012, the President of the United States made science, technology, engineering and mathematics (STEM) education a national priority by announcing an ambitious goal to increase the number of individuals who receive degrees in STEM by one million over the next decade. The President’s announcement was based on an influential report prepared by the President’s Council of Advisors on Science and Technology [1]. To achieve an increase of one million STEM graduates, institutions will need to increase the number of degrees awarded in STEM by more than 30% over current rates by 2020. Additional high profile reports from the Committee on STEM Education of the National Science and Technology Council and the National Academies of Science underscore the essential role of science and technology in the future of the United States [2,3].

Encouraging underrepresented minorities (URM) to enter STEM fields could close the STEM gap significantly as the proportion of URM students who received 4-year college degrees in STEM disciplines in 2011 (18%) is far below the proportion of minorities in the US college age population (36%) [4,5], and only 20% of URM who intend to earn a STEM undergraduate degree have done so [6, 7]. In the biological sciences, URM currently earn a combined 16% of bachelor's degrees, and this gap between the population demographics and the demographics within STEM fields is widening [4].

Although the need for degrees and diversity within STEM is widely heralded [8,9,10], the way forward to achieve these increases is not clear. In order to harness the potential of all available talent, non-traditional students should not be overlooked. Recommendations encourage 2-year and 4-year institutions to work together and form better connections among themselves to provide more entry points and pathways to STEM degrees that capture rather than exclude students [1,11,12]. Community colleges play a largely unsung, yet critical role, in the nation’s higher education system. Almost one-half of all Americans who receive a bachelor’s degree have attended a community college, and 40% of STEM graduates have attended a community college at some point in their educational career [13]. Community colleges serve the most diverse student populations in the country with a higher proportion of women, older students, first generation students, veterans, working parents, low-income students, and URM than 4-year institutions [12,13]. Many community college students have a desire to pursue a 4-year degree, but, due to a myriad of factors, these students do not complete the 2+2 pipeline, and this transfer gap is wider for URM students. For example, while nearly half of Latino community college students express an interest in transferring and earning a 4-yr degree, only 6% had earned a B.S. degree within six years [14,15]. The barriers to transfer include financial, social, and familial responsibilities [16], which prevent relocation to their state’s land-grant research university.

With the explosion of communication technologies, distance education (also called online education) continues to be a growing trend in higher education. The proportion of students who are currently taking at least one online course is 33.5% [17]. Although massive open online courses (MOOCs) have attracted great attention in recent years [18,19], students have been regularly taking online courses for over a decade and are well versed in the online learning environment. Numerous studies on the outcomes and quality of learning have shown that distance education can be as or more successful than the comparable classroom experience [20–23]. Based on an analysis of 232 studies testing the effect of distance education on learning, Bernard et al. found that asynchronous distance education significantly increases achievement and attitude but decreases retention compared to traditional classroom instruction [24]. Although statistically significant, the differences between the two methods of teaching were small leading the authors to conclude that asynchronous distance learning is equivalent in quality to face-to-face classroom learning. A second meta-analysis of 51 studies found that learning improved slightly with online instruction compared to face-to-face instruction [25]. A recent survey showed that 74% of academic leaders queried rate the learning outcomes in online education as the same or superior to those in face-to-face instruction [17].

Studies have also demonstrated success in online education in life sciences education. In one study conducted over a period of six to nine semesters, online students scored 10% higher on exams than did traditional students in an introductory microbiology course [26]. Another study showed that there was no difference in performance between students taking an introductory microbiology course in a traditional manner versus online delivery [27].

Although some studies demonstrated higher attrition rates in distance education courses compared to face-to-face courses [28], recent research shows that the gap in attrition rates between distance education and face-to-face delivery has decreased, and in fact, many asynchronously delivered courses have very high retention rates, higher than comparable face-to-face courses [29,30]. A study of the role of distance education for financially independent students (as opposed to financially dependent students) found that enrollment in distance education classes significantly decreased the likelihood of an enrollment gap among independent students [28]. Thus, online education can increase degree progress and completion rates for non-traditional undergraduates.

Responding to the need for more STEM majors required innovative approaches because, while increasing participation in STEM is essential, many institutions are at maximum physical capacity for on-campus students. The Microbiology and Cell Science Department in the College of Agricultural and Life Sciences at the University of Florida began to expand its reach through distance technology in 2011 through the establishment of a new Distance Education in Microbiology and Cell Science Bachelor of Science program (DE MCS). This program is the first distance education program for transfers from community colleges to a research university in a STEM field, and as such, this program serves as a model for other universities seeking to expand the reach of their STEM programs. To test the hypothesis that a distance education approach can successfully contribute to the STEM degree pipeline, the outcomes of the distance education program were assessed and compared to on-campus programs after three full academic years.

## Material and Methods

### Ethics statement

Most of the data used in this study are archival, aggregate data where the students have been de-identified. As a result, the work described here was viewed exempt as human subjects research by the University of Florida's Institutional Review Board. However, this analysis is a component of a larger study that was reviewed and approved by the University of Florida's Institutional Review Board (2012-U-0518). The data was provided by the University of Florida's Office of Institutional Planning and Research.

### Design of the Distance Education Program

The design of the DE MCS program was based on a framework of seven factors in developing online education programs described by Rovai and Downey [31]. These factors are planning, marketing and recruitment, financial management, quality assurance, student retention, faculty development, and course design and pedagogy.

### Program Planning

Successful online programs engage in strategic planning, which includes forming alliances with key partners that have important roles in the overall program vision [31]. Three years prior to officially launching the program, the Microbiology and Cell Science department (MCS) laid the foundation of a strong partnership with an important two-year partner in the same state, Miami Dade College (MDC). As the largest institution of higher education in Florida as well as in the United States, MDC represented a critical population of students predominantly from underrepresented backgrounds interested in earning their 4-year degree from the state’s premier research university. This collaboration led to a signed articulation agreement in 2010 between the University of Florida and Miami Dade College that allowed for a seamless transfer of MDC students interested in the distance education 2 + 2 program. Since then, similar agreements were signed with Palm Beach State College and St. Petersburg College.

### Marketing and Recruitment

Marketing and recruiting a pool of qualified students is critical for the success of a distance program [31]. In early 2011, students were recruited on an individual basis through the help of academic advisors located at the University of Florida Institute of Food and Agricultural Sciences (IFAS) Research and Education Centers. These IFAS centers are located throughout the state. In addition, early marketing efforts including advertisements in the community college newspapers and hiring enrolled students to help with peer recruiting. After the first semester, it was clear that the recruiting and marketing efforts were not sufficient to generate the numbers of applicants needed for a sustainable program. The services of a marketing firm that specializes in higher education distance program, Apollidon Learning, were retained. As recommended as a key factor of a successful distance program, the marketing team developed a program brand. The brand, logo and color scheme were consistently used in paper marketing materials and the program’s website (http://microbiology.ifas.ufl.edu).

### Financial Management

Understanding the costs associated with entering a distance education market is very important. One of the first financial decisions to be made is whether the online program will be organized as a separate division or integrated within the existing structure of the institution [31]. The DE MCS program uses a hybrid of these two approaches. Distance education programs at the University of Florida are structured so that the revenue generated from tuition and fees allows the program to be entirely self-sufficient. Although the DE MCS program has its own financial structure, it is fully integrated within the university in that the distance education (DE) students are considered full-time university students and have access to the same university resources including the library, disability resource center, counseling, etc. Some DE programs increase revenue streams by using a large pool of adjunct instructors instead of full-time faculty to teach distance students. From the start, the DE MCS program elected to use the same instructors for the DE MCS students as the on-campus students. The DE MCS students take the same departmental courses as the on-campus students. The only difference is that the DE students register through a specific section of the course for the purpose of tracking enrollment, tuition, and revenue. As it may take years before DE programs yield positive net revenues, the university provided an investment of $50,000 at the launch of the program for initial expenses.

Originally, the DE MCS program did not include a sizeable budget for marketing and recruitment, but quickly, the value of solid marketing and recruitment efforts became clear. In return for marketing and recruitment services, Apollidon Learning receives a portion of the income.

The tuition charged to the students of the DE MCS program is precisely the same level as charged to on-campus in-state students. The semester fees are also identical with the exception of small fees that only apply to on-campus students such as city/campus bus transportation fee (DE students pay about $42 less per year in fees in 2014).

### Quality Assurance

Because the DE MCS program and the traditional MCS program have the same curriculum, courses, instructors, exams, expectations, grading scales, and in many cases, the same mode of delivery, the quality of the DE MCS program should be very similar to the quality of the on-campus program (see Results). The university has a solid national reputation as a Research 1 university, but is also the flagship university in Florida, and its programs are accredited. To keep the quality as high and as consistent as possible between the DE MCS program and the on-campus program, instructors in the department have been encouraged to teach lecture courses exclusively through online delivery modes so that all students receive the same course experience. This approach also encourages the instructors to invest their time in improving the course itself rather than delivering material in multiple ways. Because the DE MCS program requires that: 1) all laboratory courses are taken in the classroom, 2) all exams are proctored, and 3) all instructors are readily available to DE students through discussion sessions or other formats, the DE MCS courses and degree are accepted by professional schools including medical and dental schools.

As a life sciences program, several laboratory courses are required for the B.S. in MCS degree including introductory biology, introductory chemistry, organic chemistry, and introductory physics with their corresponding laboratories. All of these lab courses are taken during the first two years as a community college student or at a local college with the credits accepted by the University of Florida. The MCS major has two required laboratory courses, which students take in a face-to-face format. These lab courses include an introductory microbiology lab course and an advanced lab course that gives students a more enriched lab experience. The DE MCS program has established three ways in which students can fulfill these two departmental laboratory requirements. First, if ten or more DE MCS students are located in the same region, for example near the Indian River Research and Education Center in Ft. Pierce, the department can rent teaching laboratory space to teach the laboratory courses locally. Second, students may take equivalent laboratory courses at their local institutions that will be accepted for credit by the University of Florida. Third, if the first two options are not feasible for the student, the DE MCS program has developed a creative approach to providing laboratory courses for DE students: an immersion laboratory course. Students come to the main campus for eleven days during the summer term and take the same laboratory courses as the on-campus students, but in an immersion format of several 8–10 hour days in a row. The immersion lab courses use the same curriculum and instructors as the semester-long versions of the laboratory courses. These three options provide students with ways to meet the laboratory requirements of the degree that work with their professional and personal schedules.

One recurring question from potential students, professional schools, and other interested parties is that of exam proctoring. All exams are proctored to the same extent as students who take exams in person. Depending on the course, instructor, and exam type, DE MCS students have taken exams at a regional location monitored by a paid proctor or have taken exams using commercial online proctoring services such as Remote Proctor Now from Software Secure (http://softwaresecure.com) or ProctorU (http://proctoru.com).

### Retention

Data indicates that retention rates in online programs may be lower than student retention in traditional courses [31]. Factors that may affect retention of distance students are that DE students are more likely to be nontraditional and therefore more likely to have work, family, and financial obligations than on-campus students. Additionally, DE students may experience a lack of personal connection with other students and faculty. To address retention, MCS faculty are available to online students via discussion groups, email, and social media sites like Facebook and Twitter. Students can connect with each other through course discussion pages, chats, emails, and Facebook pages. The department chair meets with DE MCS students personally or by videoconference at regional locations once each year to address any issues. Recently, the department was awarded a National Science Foundation grant from the STEM Talent Expansion Program (STEP) with our partners at MDC. This project focuses on using best practices in increasing STEM retention such as scholarships, peer tutoring, research experiences, and career mentoring to increase enrollment, retention and graduation of students from the South Florida region in the DE MCS program.

### Faculty Development

Faculty development in online teaching has been in progress since before the launch of the program. Many of the faculty development activities stemmed from faculty interested in learning best practices in online teaching from their own peers. An annual teaching symposium hosted by the College of Agricultural and Life Sciences, the teaching arm of IFAS, has sessions devoted to online teaching. The College and MCS have invested in new distance education tools, resources, and the technical support staff to ensure proper delivery of the courses and assessments. One important factor in the launch and success of this program was to have strong faculty support and interest. As all departmental courses for DE MCS students are the same as the face-to-face on-campus courses and taught by the same faculty, it was important for faculty to modify their delivery to accommodate the DE students. Faculty responded to this request with varying degrees of enthusiasm. However, faculty are encouraged to teach by distance in whichever format suits their style the best. For example, some faculty use in-class recording to record their lectures in the classroom while others use screen capture software and video podcasting for asynchronous delivery for students. Allowing faculty to choose their method for course delivery reduced the barrier of entry into the program. All faculty use the online learning management system supported through the university to host their online class. Recorded lectures are made available to on-campus as well as DE MCS students.

### Online Course Design and Pedagogy

The workshops and symposiums described above also cover online course design. One strength of the DE MCS program is that faculty are encouraged to use an online pedagogical approach that suits their needs, preferences, and design. Because instructors can utilize tools of their choice, they have more ownership and investment in the course. One unique feature of the MCS curriculum is that it integrates genomics and bioinformatics at different levels and in multiple courses. Genomics and bioinformatics are fields that integrate biology with computer science, which makes these topics a natural fit for online learning.

### Assessment of Outcomes

Student enrollment and demographic data was obtained by request from the Office of Institutional Planning and Research at the University of Florida. The enrollment data was reported as student headcounts for Microbiology and Cell Science (MCS) majors in the Fall semester from years 2009–2014 for three categories: on-campus, on-campus transfer, and distance education transfer. On-campus students are individuals who entered the institution as degree-seeking students and did not transfer from another institution or attend another higher education institution previously. On-campus transfer students began their undergraduate degree at a different institution and then transferred into the University of Florida as an MCS major. Typically, these students transfer from a 2-yr institution in the state to complete their 4-yr degree. Distance education transfers (DE MCS students) are students who transferred to the university as MCS majors, typically from a 2-yr institution, but are not physically on campus as they complete their degree in the DE MCS program.

In addition to total headcounts, the Office of Institutional Planning and Research provided the headcounts of Microbiology and Cell Science majors by self-reported Race/Ethnicity. The DE MCS program is in the College of Agricultural and Life Sciences, but students in the College of Liberal Arts and Sciences at the University of Florida also have the option to major in MCS. Although degree requirements for a B.S. in MCS are the same for all MCS majors, there are differences in requirements and expectations for students in the two colleges at the admissions level. Since the DE MCS program is only available through the College of Agricultural and Life Sciences and since the College of Liberal Arts and Sciences does not have a comparable distance education-based program, the demographics, retention, and quality of the DE MCS program is assessed by comparisons with the two most closely related cohorts: MCS on-campus (admitted as first time in college) and MCS on-campus transfers (admitted after attending another institution) from the College of Agricultural and Life Sciences.

Retention data was obtained from the Office of Institutional Planning and Research and the Office of the University Registrar. Retention was assessed in two specific transfer cohorts of Microbiology and Cell Science majors who transferred into the university as juniors in the Fall of 2011 and 2012: on-campus transfers and distance education transfers. The proportion of students from those cohorts who had graduated or were still persisting towards their B.S. degree at the two year time point (equivalent to a 4 year graduate rate) were compared.

To measure academic performance, grade point averages (GPAs) were obtained from the Office of the University Registrar and the Office of Institutional Planning and Research. Two types of GPAs were assessed for the on-campus, on-campus transfers, and DE transfers: current GPAs (as of October 2013) for all students in their junior year or above who were enrolled as MCS majors in the Fall 2013 semester and final GPAs at time of graduation for students who had graduated with a degree in MCS from Fall 2011—Spring 2014. All figures were generated with the R package, ggplot2 [32]. Qualtrics was used to develop and disseminate an anonymous and optional survey to the DE MCS students enrolled in the Fall 2014 semester.

## Results

### Enrollment

**Fig 1:**
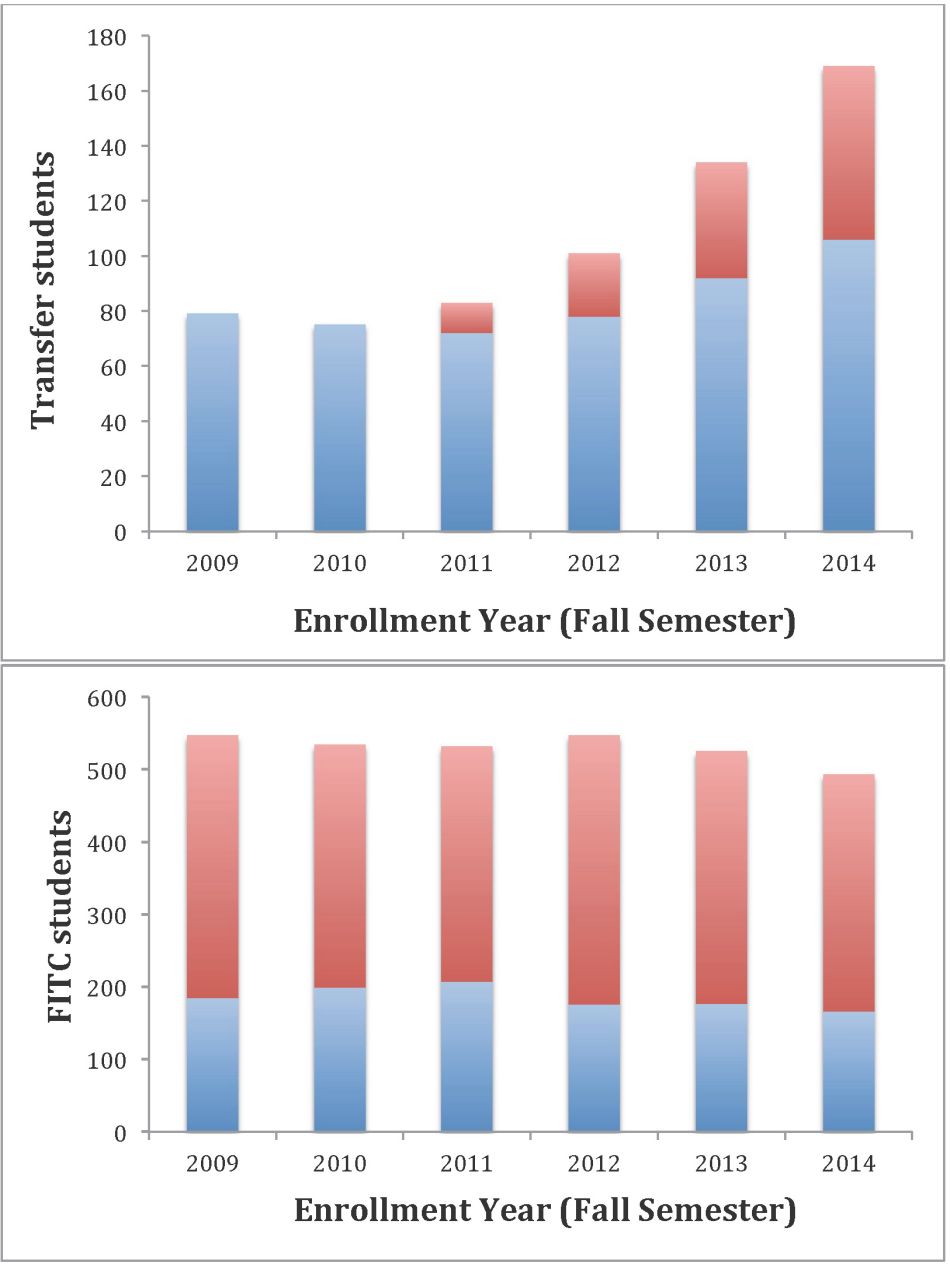
Enrollment by students into the Microbiology and Cell Science major across time. a) transfer student enrollments in the on-campus (blue) and distance education (red) and b) first time in college (FTIC) students enrolled in the College of Agricultural and Life Science (blue) or College of Liberal Arts and Sciences (red) Microbiology and Cell Science major.

The Distance Education in Microbiology and Cell Science (DE MCS) B.S. degree program is in the spring semester of its fourth year and has therefore completed three full academic years. Students are admitted and can matriculate into the program during the Fall, Spring, or Summer semesters each year. The program officially launched in Fall 2011 with 11 enrolled students and has steadily increased each year up to an enrollment of 63 students in Fall 2014 (Fig 1).

At the University of Florida (UF), students in either the College of Agricultural and Life Sciences (CALS) or the College of Liberal Arts and Sciences (CLAS) can be a Microbiology and Cell Science major. The on-campus enrollment in this major in both colleges has declined by 10% from 2009–2013 (Fig 1B). However, the total number of transfer students (both on- and off-campus) has more than doubled during that same time frame (Fig 1A), which provides a net increase in total enrollment of 5.7%.

Eighty percent of the increase in transfer students is derived from the MCS DE program. The remaining increase is from on-campus transfer students. Admission records indicate that none of the DE MCS transfer students transferred from any on-campus UF program to the DE MCS program. Hence, the DE MCS program did not take students away from any existing on-campus program. Additionally, availability of the DE MCS program caused an increase in the overall MCS enrollment since a survey of DE MCS students revealed that only 21% of current DE MCS students would have applied to an on-campus UF program if the DE MCS program were not available (see S1 Survey).

All of the above evidence supports the hypothesis that the DE MCS program increased overall enrollment in the major while having little effect on the existing on-campus programs. The surprising result is that the on-campus transfer enrollment increased along with the increase in the MCS DE program. The marketing of the DE MCS major by Apollidon may have increased the overall awareness of this MCS major statewide among community college students thereby increasing interest and eventual enrollment both off- and on-campus.

### Demographics

**Fig 2:**
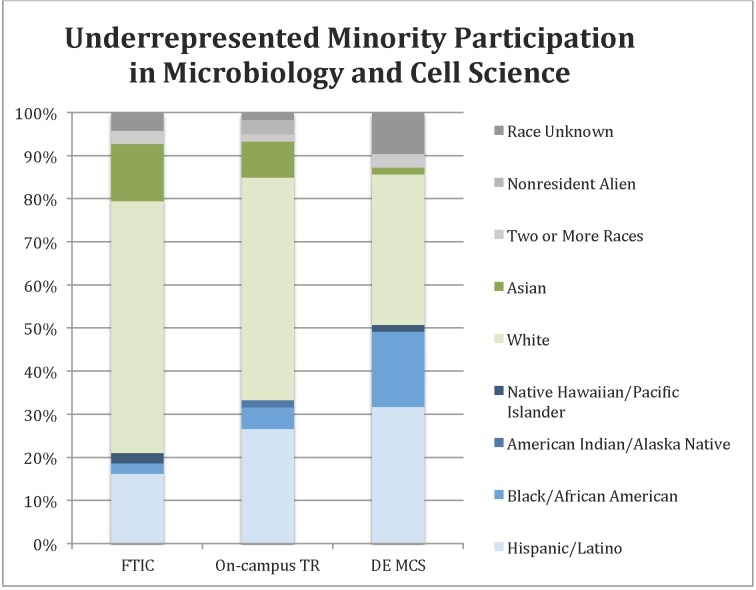
Distribution of races and ethnicities for the Fall 2014 enrollment in the Microbiology and Cell Science major within the College of Agricultural and Life Sciences. Racial/ethnic groups who are traditionally underrepresented in STEM fields are in blue while those groups who are not underrepresented in STEM are in green. The proportions of individuals with two or more races, nonresident aliens, or race unknown are represented in shades of gray.

The 2014 enrollment data indicate that the DE MSC cohort is more diverse than either the on-campus transfer students or the on-campus cohort (students admitted as first time in college) (Fig 2). These data, along with those in Fig 1, show that the DE MCS program increased overall enrollment and had the added benefit of increasing the participation of URM. According to the National Science Foundation, American Indian, Alaska Native, Black, or Hispanic ethnicities and races are traditionally underrepresented in the STEM fields [4]. The URM enrollment in the DE MCS program is 51%, which significantly exceeds the URM participation for on-campus first time in college student cohort (21%; p = 0.0001 by Fisher’s Exact Test) and on-campus transfer cohort (33%; p = 0.02 by Fisher’s Exact Test).

### Retention

As a 2+2 program, the earliest time point at which most students are in a position to graduate with their B.S. degree is two years after they transfer to the 4-year institution. Since the DE MCS program is relatively new, there are only 24 students who have been in the program long enough to reach the two-year graduation time point. To assess how the DE MCS program compares to the traditional on-campus transfer program, the graduation and persistence data from the DE-MCS students who began in the program in the Fall 2011 and Fall 2012 was compared to the corresponding on-campus transfer students who also enrolled during the same time period as MCS majors in CALS. The two cohorts of transfer students take the same MCS courses taught by the same faculty, take the same exams, and have the same requirements for graduation.

**Fig 3:**
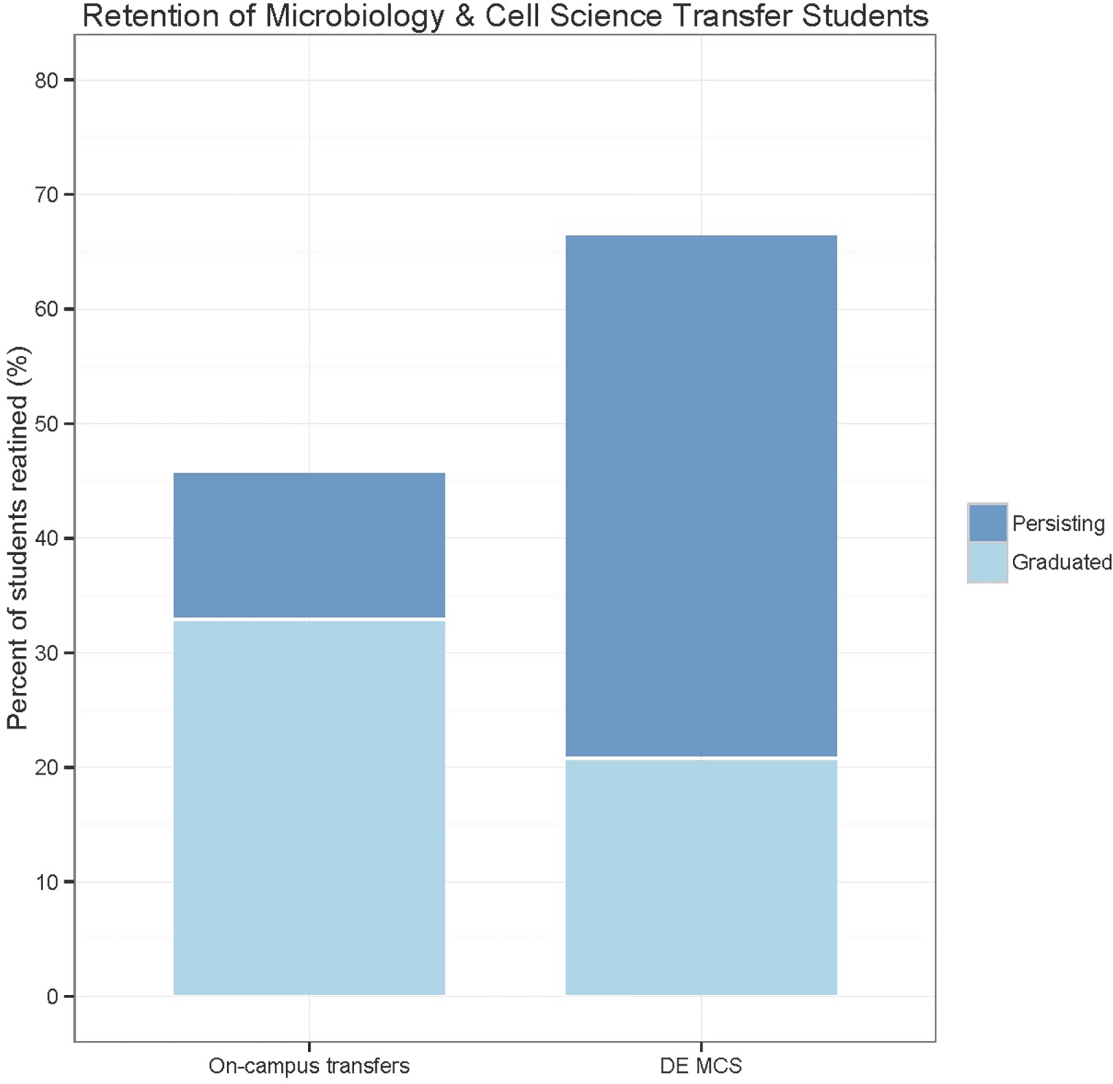
Retention of transfer students who enrolled in the Microbiology and Cell Science major within the College of Agricultural and Life Sciences in Fall 2011 and Fall 2012. Retention equals the proportion of students who have graduated and persist in the program. Retention levels between the two cohorts are not statistically different (p = 0.10, two tailed Fisher's Exact Test).

Of the 70 on-campus transfer students from this time period, 23 have graduated with their B.S. degree, which is a graduation rate of 33% (Fig 3). Of the 24 DE MCS transfer students, 5 have graduated with their B.S. degree, which is a graduation rate of 21%. Of the remaining transfer students who have not yet graduated, 13% persist in the on-campus transfer program while 46% of DE MCS students persist in the degree program. To date, the DE MCS program has a retention rate of 67%, which refers to the percent of students who have either graduated or continue to persist toward their B.S. degree. This retention rate is higher than the retention of the corresponding on-campus transfer cohort, which is 46%. As determined by Fisher's Exact Test, there is no statistical difference between the retention levels of the on-campus and distance education transfer cohorts (p = 0.10). Historically, the 4-year and 6-year graduation rates of transfer students in the on-campus Microbiology and Cell Science major are 33% and 69% respectively. Collectively, these data provide preliminary evidence that the DE MCS program is comparable to historical on-campus outcomes for transfer students, but more years of data are required for firmer conclusions.

### Academic Performance

**Fig 4:**
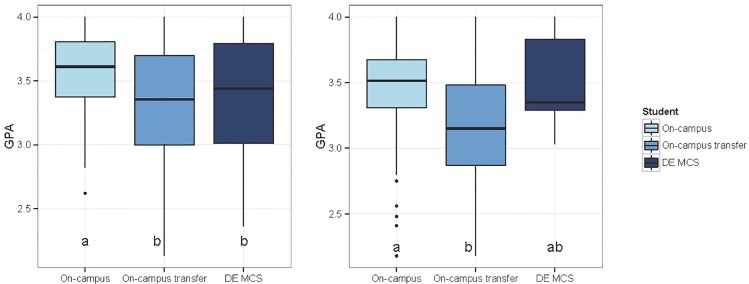
Box plots representing the average grade point average (GPA) of MCS majors within CALS. The horizontal lines represent the median GPA of students in the Fall 2013 semester (left box plot) and at the time of graduation (right box plot). The boxes represent the interquartile range (IQR). The IQR includes the 50% of samples closest to the median. The lines above and below the IQR, represent either 1.5 times the IQR or the maximum range of the samples if that range is below 1.5 times the IQR. The dots above or below these lines represent outliers that are above or below 1.5 times the IQRs. As determined by Kruskal-Wallis, the on-campus cohort had a statistically higher mean GPA than the on-campus transfer cohort (p = 0.031) but not the DE MCS cohort (p = 0.118) in the Fall 2013 semester (H = 8.5, df = 2). The mean GPAs of the two transfer cohorts were not statistically different (p = 0.956). At the time of graduation, as depicted in the right box plot, the on-campus transfer cohort had a statistically lower mean GPA than the on-campus cohort (p = 0.00016), but there was no statistical difference between the mean graduating GPAs of the on-campus and DE MCS students (p = 0.995) nor the on-campus transfer and DE MCS cohort (p = 0.269) (H = 16.5, df = 2).

As a measure of academic performance, the grade point averages (GPAs) of all CALS MCS majors in their junior year or above enrolled in Fall 2013 were collected and compared by admission type (Fig 4). Because all students enrolled in the DE MCS transfer program are in their junior or senior years, only GPAs from juniors and seniors from the on-campus and on-campus transfer cohorts were analyzed. The three cohorts take MCS courses from the same instructors and take the same exams. The two on-campus cohorts have the option to attend MCS courses in a face-to-face format, but it is not always required; however, the DE MCS cohort takes all their courses via asynchronous online learning. Beyond the courses required for the degree, students choose their own electives. The mean GPA of the Fall 2013 DE MCS students was 3.34, which was slightly higher than the mean GPA of the on-campus transfer students (Mean = 3.31), and the mean GPA of the on-campus cohort (admitted as first time in college) was 3.55. Due to the GPA scale (upper limit can not exceed 4), the GPA data was skewed and not normally distributed (Shapiro-Wilks test, p<0.05). Therefore, Kruskal-Wallis analysis with the Tukey and Kramer (Nemenyi) test (for pairwise comparisons) were applied. Using these methods, the GPA datasets demonstrated that the on-campus cohort had a statistically higher GPA than the on-campus transfers (p = 0.031) and approaching, but not significantly higher than DE MCS transfers (p = 0.118). The GPAs of the two types of transfer students were not statistically different (p = 0.956).

The final GPAs at the time of graduation were also analyzed for all CALS MCS majors who graduated between Fall 2011—Spring 2014. The three cohorts have the same requirements for graduation. The mean graduating GPAs for the on-campus cohort (admitted as first time in college) majors, on-campus transfer cohort, and DE MCS cohort were 3.45, 3.18, and 3.50, respectively (Fig 4). The mean GPA of the DE MCS graduates was comparable to both the mean GPA of the on-campus graduates and the mean GPA of the on-campus transfer graduates (p = 0.99 and p = 0.27 respectively; Kruskal-Wallis Test, H = 8.5, df = 2). The mean graduation GPA of the on-campus major is statistically higher than the mean graduation GPA of the on-campus transfers (p < 0.01; Kruskal-Wallis Test, H = 16.5, df = 2).

### Student choices and preferences

Institutional data shows that none of the currently enrolled DE MCS students transferred from any on-campus degree program or any other off-campus degree program to the DE MCS program. Thus, the DE MCS enrollment is not taking students away from any other university enrollment. In addition, a recent survey reveals that 31% of the currently enrolled DE MCS students had no other option for college. Of the 69% of DE MCS students who did have other options for college, only 30% would have transferred to an on-campus University of Florida program. These results suggest that this program is increasing STEM enrollment overall and is also increasing enrollment in STEM at the University of Florida. Survey results are provided as S1 Survey.

Valuable lessons have been learned regarding those features of the program that are appreciated by students and faculty. For example, nearly all of our students surveyed appreciated the flexibility provided by online, asynchronous lectures. Flexibility allows them to obtain a STEM education while keeping any current job schedule and maintaining family responsibilities. In addition, DE MCS students take advantage of the three methods that face-to-face lab courses are delivered as described in the Material and Methods section. The DE MCS students were nearly evenly divided in their appreciation of each approach, which indicates that each approach may have influenced enrollment. Finally, over the years, various proctoring methods have been used. The most effective and efficient way to deliver exams to the DE MCS students was to embed exams in the learning management system with ProctorU observing the student and their work on the screen. ProctorU then informs the instructor of any behavior by the students that might be construed as cheating. These issues are then investigated at the discretion of the instructor.

## Discussion

To answer the national call for more individuals with STEM degrees while increasing diversity in the STEM fields, the Microbiology and Cell Science Department in the College of Agricultural and Life Sciences at the University of Florida developed a new type of a 2+2 distance education program. This program is the first of its kind: a distance education-based transfer program in a life science offered by a research-intensive university. Data and a student survey from the initial years of this program suggest that this program increases enrollment in Microbiology and Cell Science without drawing from existing on-campus programs and that distance education students were more diverse, had a similar retention level, and comparable GPAs than on-campus transfer students. Larger sample sizes and more years of student participation in the program will provide a deeper analysis as the program progresses.

A recent report emphasizes the importance of increasing the participation of underrepresented minorities (URM) in the STEM education pipeline, and one recommendation to accomplish this is to establish strategic and strong partnerships [1]. As community colleges are lower in cost and geographically accessible, underrepresented minorities attend these institutions at higher rates than 4-yr institutions [13]. These two factors, lower cost and geographic accessibility are critical in increasing the STEM degree attainment of less economically advantaged Hispanic students [33]. Community colleges play a very important role in undergraduate biology education and cooperation between 2-yr colleges and universities is essential in developing opportunities for undergraduates [34].

Half of the currently enrolled DE MCS students are individuals from race/ethnic backgrounds underrepresented in STEM. From this aspect, the DE MCS program is a notable success in increasing URM participation in STEM. The URM proportion is significantly higher in the DE MCS program than in the comparable on-campus program.

Retention and academic performance were measured to assess the quality of the DE MCS program. Although the numbers are small, the outcomes are promising. The retention for the DE MCS program was 67% and not statistically different from the on-campus cohort. A recent report from the National Student Clearinghouse cites that four years after transferring to a 4-year institution, 72% of transfer students (originating at a 2-yr institution) have either graduated or are persisting in degree attainment [35]. This statistic is for all degrees, not just for STEM, but indicates that DE MCS program is on par with published retention rates for transfer students. Retention in STEM is a significant concern as fewer than 40% of students who enter college intending to major in STEM finish with a 4-year STEM degree [1]. Although this program targets students who have already completed the first two years of their degree, lessons learned from this model can influence STEM retention.

The data presented here indicates that the DE MCS at the University of Florida is succeeding as a viable approach to increasing STEM participation and diversity without pulling students away from existing on- or off-campus programs. The hybrid, asynchronous format allows students the opportunity to achieve their STEM degree while, in many cases, working full-time and raising children. The data indicates that students in the DE MCS program are receiving an education that is as good as its on-campus counterpart with similar retention levels and similar grade point averages. Because it was structured to be as similar to the on-campus programs as possible, students receive essentially the same education and have the same post-baccalaureate opportunities including acceptance into graduate and health professional schools. As the DE MCS program continues to grow, future studies will capture additional data including student perceptions, long-term outcomes, the role of scholarships, research experiences, and tutoring on student performance, and predictors of success. Just as innovation and creativity are vital to maintain the nation’s STEM preeminence, which is essential to the economy, healthcare, environment, and security of all citizens, innovation and creativity are also needed to address the gap in STEM education.

With its unique hybrid online approach, the Distance Education in Microbiology and Cell Science Bachelor of Science program represents a new type of transfer pipeline that leverages the curriculum of a land-grant university and online learning technologies to overcome geographical and financial barriers that may prevent underrepresented minorities from achieving their B.S. degree in a STEM field. In an era of ever-increasing costs, this program demonstrates another route by which land-grant universities can fulfill their mission to provide access to affordable public higher education.

## Supporting Information

S1 Dataset Enrollment, retention, and GPAs. Datasets that were used in analysis of enrollment, retention, and academic performance of Distance Education in Microbiology and Cell Science Majors
(XLSX)

S1 Survey Survey to Distance Education in Microbiology and Cell Science Majors. Survey administered anonymously to students currently enrolled in the Distance Education in Microbiology and Cell Science major.
(PDF)

## References

[1] Olson S, Riordan DG. Engage to excel: producing one million additional college graduates with degrees in science, technology, engineering, and mathematics. Report to the President. Executive Office of the President. 2012. Available: http://files.eric.ed.gov/fulltext/ED541511.pdf

[2] Federal Science, Technology, Engineering, and Mathematics (STEM) Education 5-Year Strategic Plan. A Report from the Committee on STEM Education. National Science and Technology Council. Executive Office of the President. 2013. Available: http://www.whitehouse.gov/sites/default/files/microsites/ostp/stem_stratplan_2013.pdf

[3] National Academy of Sciences, National Academy of Engineering, and Institute of Medicine. Rising Above the Gathering Storm, Revisited: Rapidly Approaching Category.5 Washington, DC: The National Academies Press; 2010 Available: http://www.nap.edu/catalog/12999/rising-above-the-gathering-storm-revisited-rapidly-approaching-category-5 25506949

[4] National Science Board. Science and Engineering Indicators. Arlington (VA): National Science Foundation NSB 14–01. 2014 Available: http://www.nsf.gov/statistics/seind14/

[5] U.S. Census Bureau, Census; 2010.American FactFinder. Database: American Factfinder [Internet]. Available: http://factfinder2.census.gov Accessed 2014 May 20.

[6] National Research Council and National Academy of Engineering. Community colleges in the evolving STEM education landscape: Summary of a summit Washington DC: National Academies Press; 2012 Available: http://www.nap.edu/catalog/13399/community-colleges-in-the-evolving-stem-education-landscape-summary-of

[7] National Academy of Sciences, National Academy of Engineering, and Institute of Medicine. Expanding Underrepresented Minority Participation: America’s Science and Technology Talent at the Crossroads. Washington DC: National Academies Press; 2011 Available: http://www.nap.edu/catalog/12984/expanding-underrepresented-minority-participation-americas-science-and-technology-talent-at 10.1097/ACM.0b013e3181b6c76b.The 22379652

[8] Antonio AL, Chang MJ, Hakuta K, Kenny DA, Levin S, Milem JF. Effects of racial diversity on complex thinking in college students. Psychol Sci. 2004; 15: 507–510. 15270993 10.1111/j.0956-7976.2004.00710.x

[9] Page SE. The difference: how the power of diversity creates better groups, firms, schools, and societies Princeton (NJ): Princeton University Press; 2007.

[10] Maltese AV, Tai RH. Pipeline persistence: examining the association of educational experiences with earned degrees in STEM among US students. Sci Educ. 2011; 95: 877–907.

[11] National Research Council. Rising above the gathering storm two years later: accelerating progress toward a brighter economic future summary of a convocation. Washington, DC: The National Academies Press; 2009 Available: http://www.nap.edu/catalog/12537/rising-above-the-gathering-storm-two-years-later-accelerating-progress

[12] Labov JB. Changing and evolving relationships between two- and four-year colleges and universities: they’re not your parents’ community colleges anymore. Cell Biol Educ. 2012; 11: 121–128. 10.1187/cbe.12-03-0031 PMC3366895 22665585

[13] Tsapogas. The Role of community colleges in the education of recent science and engineering graduates Arlington (VA): National Science Foundation, Division of Science Resource Statistics NSF 04–315. 2004.

[14] Nuñez A-M, Sparks J, Hernández E. Latino access to community colleges and Hispanic-Serving institutions. J Hispanic High Educ. 2011; 8(4): 322–339.

[15] Radford AW, Berkner L, Wheeless SC, Shepherd B. Persistence and attainment of 2003–04 beginning postsecondary students: after 6 years Washington DC: US Department of Education, National Center for Education Statistics NCES 2011–11. 2010.

[16] Nuñez A-M, Elizondo D. Closing the Latino/a transfer gap: Creating Pathways to the Baccalaureate. Perspectivas: Issues in Higher Education Policy and Practice 2013; 2.

[17] Allen IE, Seaman J. Grade change: tracking online education in the United States Babson Survey Research Group and Quahog Research Group; 2014.

[18] Bidwell A. Where Do MOOCs fit in higher education. US News & World Report. 2014 May 15. Available: http://www.usnews.com/news/articles/2014/05/15/where-do-moocs-fit-in-higher-education

[19] Wasik JF. What color is your online adult course? The New York Times. 2014 March 18. Available: http://www.nytimes.com/2014/03/18/education/what-color-is-your-online-adult-course.html

[20] Dell CA, Low C, Wilker JF. Comparing student achievement in online and face-to-face class formats. J Online Learn Teach. 2010; 6: 30–42.

[21] Weber JM, Lennon R. Multi-course comparison of traditional versus web-based course delivery systems. Journal of Educators Online 2007; 4 (2): 1–19.

[22] Warren LL, Holloman HL. On-line instruction: are the outcomes the same? Journal of Instructional Psychology 2005; 32: 148–151.

[23] Lack KA. Current status of research on online learning in postsecondary education. Ithaka S+ R 2013. Available: http://sr.ithaka.org/research-publications/current-status-research-online-learning-postsecondary-education

[24] Bernard RM, Abrami PC, Lou Y, Borokhovski E, Wade A, Wozny L et al How does distance education compare with classroom instruction? A meta-analysis of the empirical literature. Rev Educ Res. 2004; 74: 379–439.

[25] Office of Planning, Evaluation, and Policy Development. Evaluation of Evidence-Based Practices in Online Learning: A Meta-Analysis and Review of Online Learning Studies. Washington, DC: U.S. Department of Education 2009.

[26] Alisauskas R. “The Love Triangle” forging links to students using digital technology to deliver content in microbiology courses. Focus on Microbiol. Educ. 2007; 13: 13–15.

[27] Jones LWK. Experiences vary in learning microbiology online. Microbe Wash DC. 2010; 5: 520–525.

[28] Pontes MCF, Pontes NMH. Enrollment in distance education classes is associated with fewer enrollment gaps among independent undergraduate students in the US. Online Learning (formerlyJournal of Asynchronous Learning Networks). 2012; 16 (1): 79.

[29] Meyer KA, Bruwelheide J, Poulin R. Why they stayed: near-perfect retention in an online certification program in library media. Online Learning (formerly Journal of Asynchronous Learning Networks. 2009; 13(3): 129–145.

[30] Moore JC, Sener J, Fetzner M. Getting better: ALN and student success. Online Learning (formerly Journal of Asynchronous Learning Networks). 2009; 13(3): 85–114.

[31] Rovai AP, Downey JR. Why some distance education programs fail while others succeed in a global environment. The Internet and Higher Education. 2010; 13: 141–147.

[32] Wickham H. ggplot2 Elegant graphics for data analysis New York (NY): Springer; 2009.

[33] Pérez PA, McDonough PM. Understanding Latina and Latino college choice a social capital and chain migration analysis. J Hispanic High Educ. 2008; 7: 249–265.

[34] Fletcher LA, Carter VC. The important role of community colleges in undergraduate biology education. CBE Life Sci Educ. 2010; 9: 382–383. 10.1187/cbe.10-09-0112 21123677 PMC2995748

[35] National Student Clearinghouse Research Center. Snapshot Report Degree Attanment: Outcomes of students who transferred from two-year to four-year institutions. National Student Clearinghouse. 2012. Available: http://nscresearchcenter.org/snapshotreport-degreeattainment2/#more-966

