# Supplementary material for: Development of a Distance Education Program by a Land-Grant University Augments the 2-Year to 4-Year STEM Pipeline and Increases Diversity in STEM
Source: PLoS One. 2015 Apr 15;10(4):e0119548. doi: 10.1371/journal.pone.0119548 (PMC4398396; doi:10.1371/journal.pone.0119548)
Supplement: S1 Survey — Survey administered anonymously to students currently enrolled in the Distance Education in Microbiology and Cell Science major. (PDF) [file pone.0119548.s002.pdf]

Use the scale below to indicate how much you agree or disagree with each of the following statements to explain your reasons for enrolling in the Distance Education in Microbiology and Cell Science B.S. program:

#	Question	Strongly Disagree	Disagree	Neither Agree nor Disagree	Agree	Strongly Agree	Total Responses	Mean
1	I preferred the flexibility of the online format.	0	2	2	3	18	25	4.48
2	The online program was more affordable than relocating to Gainesville.	1	1	4	4	15	25	4.24
3	The online program allowed me to keep my current job.	0	0	4	4	17	25	4.52
4	It is difficult for me to relocate to Gainesville because of personal responsibilities/commitments in my home area (family, dependents)	0	1	2	10	12	25	4.32
5	Other - explain below.	1	0	1	1	1	4	3.25

Other - explain below.
I don't have to pay for daycare to attend school.
I learn better by research and reading and not lectures therefore the on-line environment suits me well.

Statistic	I preferred the flexibility of the online format.	The online program was more affordable than relocating to Gainesville.	The online program allowed me to keep my current job.	It is difficult for me to relocate to Gainesville because of personal responsibilities/commitments in my home area (family, dependents)	Other - explain below.
Min Value	2	1	3	2	1
Max Value	5	5	5	5	5
Mean	4.48	4.24	4.52	4.32	3.25
Variance	0.93	1.27	0.59	0.64	2.92
Standard Deviation	0.96	1.13	0.77	0.80	1.71
Total Responses	25	25	25	25	4

Please indicate which of the following you were considering or not considering at the time you enrolled in the Distance Education Microbiology and Cell Science B.S. program. If you were not eligible for any of the options please select N/A:

#	Question	Yes - was considering	No - was not considering	N/A	Total Responses	Mean
1	Enrolling in another B.S. degree program as a transfer student	20	9	1	30	1.37
3	Working	22	3	3	28	1.32
4	Working and taking courses as a non degree student	9	17	2	28	1.75
5	Not working and not attending school	0	21	5	26	2.19
6	Other. Please specify:	1	1	3	5	2.40

Other. Please specify:
Combo programs.

Statistic	Enrolling in another B.S. degree program as a transfer student	Working	Working and taking courses as a non degree student	Not working and not attending school	Other. Please specify:
Min Value	1	1	1	2	1
Max Value	3	3	3	3	3
Mean	1.37	1.32	1.75	2.19	2.40
Variance	0.31	0.45	0.34	0.16	0.80
Standard Deviation	0.56	0.67	0.59	0.40	0.89
Total Responses	30	28	28	26	5

You indicated you were considering enrolling in another B.S. degree program as a transfer student at the time you enrolled in the Distance Education Microbiology and Cell Science program. Please indicate the field of the program you were considering the most strongly:

#	Answer		Response	%
1	Microbiology		3	15%
2	Another life science field such as Biology/Genetics/Ecology (but not microbiology)		15	75%
3	Another science, technology, engineering or math field (but not a life science)		1	5%
4	A non-science field		0	0%
5	Other		1	5%
	Total		20	100%

Other
Nursing

Statistic	Value
Min Value	1
Max Value	5
Mean	2.05
Variance	0.68
Standard Deviation	0.83
Total Responses	20

You indicated you were considering enrolling in another B.S. degree program as a transfer student at the time you enrolled in the Distance Education Microbiology and Cell Science program. Please indicate the type of institution that you were considering enrolling in the most strongly:

#	Answer		Response	%
1	University of Florida (main campus)		6	30%
2	large, research university such as Florida International University or University of Central Florida		7	35%
3	Public Florida College such as Miami Dade College, Valencia College, Palm Beach College		4	20%
4	An online program. Please specific which one(s)		0	0%
5	Other		3	15%
	Total		20	100%

An online program. Please specific which one(s)	Other
	University of Tampa or University of South Florida
	UMiami

Statistic	Value
Min Value	1
Max Value	5
Mean	2.35
Variance	1.82
Standard Deviation	1.35
Total Responses	20

MCB 3023 - Principles of Microbiology

#	Question	Have taken/ plan to take	Preferred format	Total Responses
1	Lab taught by UF faculty at a regional teaching laboratory such as the IFAS Indian River and Ft. Lauderdale Research and Education Centers	14	8	22
2	Lab taught by UF faculty at the main campus in Gainesville in a 9-day intensive format ("bootcamp" lab)	12	10	22
3	Comparable lab course taken at another institution and transferred for credit	9	5	14

Statistic	Lab taught by UF faculty at a regional teaching laboratory such as the IFAS Indian River and Ft. Lauderdale Research and Education Centers	Lab taught by UF faculty at the main campus in Gainesville in a 9-day intensive format ("bootcamp" lab)	Comparable lab course taken at another institution and transferred for credit
Min Value	1	1	1
Max Value	2	2	2
Total Responses	17	16	13

MCB 4034 - Advanced Microbiology Lab

#	Question	Have taken/plan to take	Preferred format	Total Responses
1	Lab taught by UF faculty at a regional teaching laboratory such as the IFAS Indian River and Ft. Lauderdale Research and Education Centers	11	8	19
2	Lab taught by UF faculty at the main campus in Gainesville in a 9-day intensive format ("bootcamp" lab)	13	9	22
3	Comparable lab course taken at another institution and transferred for credit	5	5	10

Statistic	Lab taught by UF faculty at a regional teaching laboratory such as the IFAS Indian River and Ft. Lauderdale Research and Education Centers	Lab taught by UF faculty at the main campus in Gainesville in a 9-day intensive format ("bootcamp" lab)	Comparable lab course taken at another institution and transferred for credit
Min Value	1	1	1
Max Value	2	2	2
Total Responses	14	16	10

Please use scale below to indicate how valuable or invaluable you consider each of the following elements

of the Distance Education Microbiology and Cell Science Program to be for your educational experience:							
#	Question	Not Valuable	Slightly Valuable	Valuable	Very Valuable	Total Responses	Mean
1	Having the options to take the lab courses in three different ways	2	0	11	15	28	3.39
2	Taking the laboratory courses in a face to face format	0	2	5	20	27	3.67
3	Learning the material through web-based lectures (lectures created directly for online learning)	1	4	9	15	29	3.31
4	Learning the material through recordings of live lectures (recordings of an instructor giving a lecture in a classroom)	3	3	6	15	27	3.22
5	Asynchronous format of courses (viewing lectures/materials on your own schedule instead of a set class time)	1	1	5	20	27	3.63
6	Remote proctoring of exams (ProctorU/Remote Proctor)	0	5	8	13	26	3.31
7	Communication with instructors	2	6	7	11	26	3.04

Statistic	Having the options to take the lab courses in three different ways	Taking the laboratory courses in a face to face format	Learning the material through web-based lectures (lectures created directly for online learning)	Learning the material through recordings of live lectures (recordings of an instructor or giving a lecture in a classroom)	Asynchronous format of courses (viewing lectures/materials on your own schedule instead of a set class time)	Remote proctoring of exams (ProctorU/Remote Proctor)	Communication with instructors
Min Value	1	2	1	1	1	2	1
Max Value	4	4	4	4	4	4	4
Mean	3.39	3.67	3.31	3.22	3.63	3.31	3.04
Variance	0.69	0.38	0.72	1.10	0.55	0.62	1.00
Standard Deviation	0.83	0.62	0.85	1.05	0.74	0.79	1.00
Total Responses	28	27	29	27	27	26	26

How did you first become aware of the Distance Education Microbiology and Cell Science B.S. program at UF?

#	Answer		Response	%
1	Career/Student activity fair		3	10%
2	Online advertisement		5	17%
3	An advisor		4	13%
4	A fellow student or friend		9	30%
5	An email from UF		1	3%
6	Other - please specify.		8	27%
	Total		30	100%

Other - please specify.
Flyer at IRSC
goggle search
TC Palm Newspaper ad in the Classified section
Searching UF website for on-line degree programs
Palm Beach State had a poster displayed around their school
Saw a flyer at Palm Beach State College
UF website
searching through UF degree programs online

Statistic	Value
Min Value	1
Max Value	6
Mean	3.80
Variance	2.86
Standard Deviation	1.69
Total Responses	30
